# Therapeutic strategy in psoriatic arthritis: choosing the right treatment for the right patient

**DOI:** 10.1177/1759720X251383084

**Published:** 2025-10-01

**Authors:** George E. Fragoulis, Sizheng Steven Zhao

**Affiliations:** First Department of Propedeutic and Internal Medicine, National and Kapodistrian University of Athens, 17 Agiou Thoma Street, Athens 10679, Greece; Centre for Musculoskeletal Research, Division of Musculoskeletal and Dermatological Science, School of Biological Sciences, Faculty of Biological Medicine and Health, The University of Manchester, Manchester Academic Health Science Centre, Manchester, UK; NIHR Manchester Biomedical Research Centre, Manchester University NHS Foundation Trust, Manchester, UK

**Keywords:** psoriatic arthritis, therapeutic strategy

Psoriatic arthritis (PsA) is a heterogeneous inflammatory arthritis characterized not only by musculoskeletal involvement but also by a substantial burden of extra-musculoskeletal manifestations and multisystem comorbidity.^1,2^ A therapeutic focus limited to musculoskeletal symptoms risks neglecting the principal drivers of long-term morbidity and mortality—cardiometabolic and mental health conditions. This supplement brings together a series of articles that reflect the evolving understanding of PsA pathogenesis and its diverse clinical manifestations while highlighting the need for a more integrated, comorbidity-focused approach to treatment.

Our understanding of the pathogenetic mechanisms underlying PsA continues to improve. The major role of IL-23 and IL-17 is well described,^3^ while various factors, including genetic features, mechanical stress, and microbiome, have been recognized to contribute to the initiation and perpetuation of the disease.^4,5^ In line with other investigators, Liu et al.^6^ review data demonstrating differences in the gut microbiome between people with PsA and healthy individuals. This adds to the evidence base for a potential link between the gut and the joint in the pathogenesis of PsA. In fact, it has been hypothesized that alterations in the gut microbiome might lead to IL-23 production, which, in turn, activates IL-23-responsive cells (e.g., Mucosal-associated invariant T cell – MAIT cells) that can migrate to the joints.^7^

Improved understanding of PsA pathogenesis has led to the development of novel therapeutic strategies. Inhibitors of IL-17, IL-23, and, more recently, JAK have expanded the therapeutic armamentarium. However, given the clinical complexity and heterogeneity of PsA, selecting the most appropriate treatment remains challenging.^8^ The recent European Alliance of Associations for Rheumatology (EULAR) recommendations place comorbidities at the center of therapeutic decision-making in PsA. Among these, cardiometabolic comorbidities are both common and a leading cause of mortality.^9^ Hypertension, for example, is reported in more than 30% of PsA patients,^1^ while in some real-world large cohorts, this figure exceeds 40%.^10^ The elevated cardiovascular (CV) risk is not solely due to the higher prevalence of traditional CV risk factors—such as obesity, smoking, and dyslipidemia. Persistent systemic inflammation also exerts deleterious effects on tissues, including the liver, vasculature, and reticuloendothelial system.^11^

Effective treatment of PsA with targeted anti-rheumatic therapies and consequent reduction in systemic inflammation may offer CV protection. While such protective effects are well established in rheumatoid arthritis,^12^ further data are needed to confirm similar outcomes in PsA. Evidence derived from psoriasis studies suggests that TNFi may reduce CV risk.^13^ However, the impact of other therapeutic classes on CV outcomes in PsA remains inconclusive, with existing data yielding conflicting results.^14,15^

Adding further complexity, the severity of psoriasis appears to correlate with an increased risk of CV disease, suggesting that differential efficacy of biologic Disease modifying anti-rheumatic drugs (bDMARDs) on skin manifestations may influence CV outcomes. Moreover, given the association between JAK inhibitors (JAKi) and thromboembolic events in Rheumatoid arthritis (RA), the EULAR guidelines advise careful consideration of CV risk factors when prescribing JAKi.^16^ Supporting this, a recent study by Colaco et al.^17^ emphasized the importance of traditional CV risk factors—such as hypertension and obesity—in predicting CV events in PsA patients.

Metabolic syndrome (MetS) is also highly prevalent among individuals with PsA, further contributing to CV risk. A recent systematic literature review (SLR) estimated MetS prevalence ranging from 23.5% to 62.9% in this population.^18^ As detailed in the comprehensive review by Williams et al.,^19^ MetS has far-reaching implications for both disease pathogenesis and treatment. Obesity, a core component of MetS, is especially relevant in PsA, with approximately one-third of patients affected in real-world studies.^10^

Not only is obesity a risk factor for the development of PsA in healthy individuals and those with psoriasis,^20^ but it is also associated with poorer long-term treatment outcomes.^21^ Notably, the negative impact of obesity appears more pronounced in patients treated with TNFi, whereas it is minimal or absent in those receiving IL-17i or IL-23i, as reported in a recent SLR.^22^

Alongside metabolic factors, mental health disorders are also highly prevalent in PsA, influencing both patient outcomes and treatment decisions. In an SLR and meta-analysis, mild and moderate depression were detected in 20% and 14% of the patients, respectively, while the percentages for anxiety were 33% for mild and 21% for moderate. Although one should avoid certain drugs (i.e., apremilast and brodalumab),^2^ there are no studies suggesting that one therapeutic approach is better than the other in patients having anxiety/depression as comorbid conditions.

Infections represent another important comorbidity, often regarded as an inevitable adverse effect of bDMARD use in PsA. According to a review by Vassilopoulos et al.^23^ in this issue of *TAMD*, the incidence of infections does not differ significantly across drug classes, although IL-23 inhibitors may offer a more favorable safety profile regarding serious infections. As regards csDMARDs, data so far support that the risk for infections is not increased in patients treated with methotrexate. For chronic and opportunistic infections, the EULAR recommendations advise routine screening for tuberculosis, hepatitis B, and hepatitis C prior to initiating bDMARD therapy.^24^ A recent SLR and meta-analysis confirmed that the risk of hepatitis B virus reactivation for IL-17, IL-23, and JAKi is comparable to that seen with TNF-inhibitors,^25^ while the risk of tuberculosis appears to be lower with IL-17i and IL-23i compared to other agents, though additional data are needed.^23^

Despite advances in treatment, minimal disease activity is achieved in only approximately one-third of PsA patients.^26^ This reflects the need for a more tailored approach in the treatment of PsA. While awaiting the development of biomarkers, so far, the choice of treatment is based on clinical, laboratory, and imaging characteristics. Demographics possibly also play a role, as it seems that there are differences between men and women in the clinical expression as well as in the treatment response.^27^ Notably, sex and gender differences may influence not only clinical presentation and treatment response but also comorbidity profiles in PsA. For example, cardiometabolic comorbidities appear more prevalent in men, whereas women more frequently report anxiety and depression—factors that may shape therapeutic priorities and outcomes. Understanding these differences could support more individualized treatment strategies.

To better characterize the disease, several new concepts have emerged. The term “preclinical PsA” has been introduced to describe psoriasis patients presenting with arthralgia,^28^ in parallel with the concept of “pre-RA” in rheumatoid arthritis. However, the optimal timing and intensity of treatment in such cases remain unclear. Early imaging may help identify patients at risk of progression and guide appropriate therapeutic interventions.

Another evolving concept is that of “difficult-to-treat,” “difficult-to-manage,” or “refractory” PsA. Although definitions are pending, this terminology aims to classify patients who, despite advanced therapy with bDMARDs, fail to achieve satisfactory clinical outcomes. For such patients, the therapeutic landscape may be shifting toward combination regimens involving two bDMARDs or the use of bispecific monoclonal antibodies.

Furthermore, as discussed in the review by Williams et al.,^19^ the emergence of novel anti-obesity agents—such as glucagon-like peptide-1 receptor agonists and sodium–glucose cotransporter-2 inhibitors—may reshape the treatment paradigm for PsA. The combination of these agents with bDMARDs has the potential to optimize clinical outcomes.

In conclusion, the contributions in this supplement underscore the growing imperative to manage PsA as a multisystem disorder—one in which cardiometabolic health, mental well-being, and infection risk are as critical as joint control. Advances in pathogenesis have opened the door to novel therapeutic approaches, yet a substantial proportion of patients fail to achieve optimal outcomes. Precision strategies that account for comorbidities, biological endotypes, and demographic variability are needed to move beyond current treatment paradigms. As the field continues to evolve, integrating rheumatologic, metabolic, and psychosocial care will be essential to improving long-term outcomes for individuals living with PsA.

## Declarations

**Ethics approval and consent to participate:** Not applicable.

**Consent for publication:** Not applicable.

**Availability of data and materials:** Not applicable.
